# *Zygosaccharomyces bailii* Is a Potential Producer of Various Flavor Compounds in Chinese *Maotai*-Flavor Liquor Fermentation

**DOI:** 10.3389/fmicb.2017.02609

**Published:** 2017-12-22

**Authors:** Yan Xu, Yan Zhi, Qun Wu, Rubing Du, Yan Xu

**Affiliations:** State Key Laboratory of Food Science and Technology, Key Laboratory of Industrial Biotechnology, Ministry of Education, Synergetic Innovation Center of Food Safety and Nutrition, School of Biotechnology, Jiangnan University, Wuxi, China

**Keywords:** *Zygosaccharomyces bailii*, food fermentation, flavor compounds, genome, transcriptome, Chinese *Maotai*-flavor liquor

## Abstract

*Zygosaccharomyces bailii* is a common yeast in various food fermentations. Understanding the metabolic properties and genetic mechanisms of *Z. bailii* is important for its industrial applications. Fermentation characteristics of *Z. bailii* MT15 from Chinese *Maotai*-flavor liquor fermentation were studied. *Z. bailii* MT15 produced various flavor compounds, including 19 alcohols, six acids, three esters, three ketones, and two aldehydes. Moreover, production of acids and aldehydes were increased by 110 and 41%, respectively, at 37°C (the maximum temperature in liquor fermentation) compared with that at 30°C, indicating its excellent flavor productivity. *Z. bailii* MT15 is a diploid with genome size of 20.19 Mb. Comparative transcriptome analysis revealed that 12 genes related to amino acid transport were significantly up-regulated (2.41- to 5.11-fold) at 37°C. Moreover, genes *ARO8, ARO9*, and *ALDH4* involved in amino acid metabolism also showed higher expression levels (>1.71-fold) at 37°C. Increased substrate supply and a vigorous metabolism might be beneficial for the increased production of acids and aldehydes at 37°C. This work revealed the potential contribution of *Z. bailii* to various flavor compounds in food fermentation, and produced insights into the metabolic mechanisms of *Z. bailii* in flavor production.

## Introduction

*Zygosaccharomyces bailii* is a yeast species widely present in various food fermentations, such as wine, tea, and vinegar fermentations (Teoh et al., 2004; Solieri et al., 2006; Garavaglia et al., 2015). In most food and beverage industries, *Z. bailii* is considered as a problematic spoilage yeast due to its high resistance to preservatives and high tolerance of various stresses (Stratford et al., 2013; Palma et al., 2015). Regardless of its association with spoilage, the potential beneficial effects of *Z. bailii* have also been proposed in food industries (Ciani et al., 2009; Domizio et al., 2011). In wine fermentation, owing to its high production of esters, *Z. bailii* in a mixed starter with *Saccharomyces cerevisiae* improved the production of ethyl esters (Garavaglia et al., 2015). The co-culture of *Z. bailii* with *S. cerevisiae* also increased the production of polysaccharides that improved the taste and body of wine (Domizio et al., 2011). Additionally, *Z. bailii* formed part of the tea fungus in Kombucha and Haipao tea fermentation that were rich in crude protein, crude fiber, and lysine (Jayabalan et al., 2010). Nevertheless, the metabolic properties and genetic mechanisms of *Z. bailii* in food fermentations are still unclear and need to be systematically studied.

Chinese *Maotai*-flavor liquor is a popular alcoholic beverage even in other parts of the world (Xu and Ji, 2012). This liquor contains over 300 influential flavor compounds including alcohols, acids, esters, ketones, and aldehydes, which greatly contribute to its unique aroma and quality (Xu and Ji, 2012). *Maotai*-flavor liquor is produced from grains by a spontaneous and solid-state fermentation. Yeasts play essential roles in *Maotai*-flavor liquor fermentation (Wu, 2013; Wu et al., 2013). Among them, *S. cerevisiae* is one of the most important and contributes significantly to the quantity and quality of the liquor (Wu et al., 2012; Meng et al., 2015). *Z. bailii* has been found to be a dominant species in *Maotai*-flavor liquor fermentation, with proportions close to those of *S. cerevisiae* (Wu, 2013). Despite its large population, the metabolic activity of *Z. bailii* in the liquor fermentation is still unclear.

The present work is aimed to study the fermentation characteristics of *Z. bailii* MT15, including the genome sequencing and comparative transcriptome analysis to unravel its metabolic mechanisms at the relatively high temperature used in liquor fermentation. The results shed new light on the function of *Z. bailii* and provide a guide for its efficient use in food fermentation.

## Materials and methods

### Yeast strains

*Z. bailii* MT15 and *S. cerevisiae* MT1 were previously isolated from the *Maotai*-flavor liquor fermentation process and were deposited in the China General Microbiological Culture Collection Center with accession number CGMCC 4745 (Xu et al., 2013), and the China Center for Type Culture Collection with accession number CCTCC M2014463 (Meng et al., 2015), respectively.

### Fermentation conditions

Sorghum extract was used as fermentation medium and was prepared according to the method below. Two kilogram of ground sorghum was added to 8 L of deionized water. Then the mixture was steamed for 2 h and subsequently saccharified by glucoamylase (5 U/L) at 60°C for 4 h. The supernatant was collected after being filtered through gauze and centrifuged at 8,000 × *g* for 15 min. The obtained sorghum extract was diluted with water to produce a final reducing sugar concentration of 75 ± 5 g/L before sterilization (Lu et al., 2015). Fermentation media were prepared with aliquots of 50 mL sorghum extract in 250 mL flasks, and were sterilized at 115°C for 30 min.

**Figure 1:**
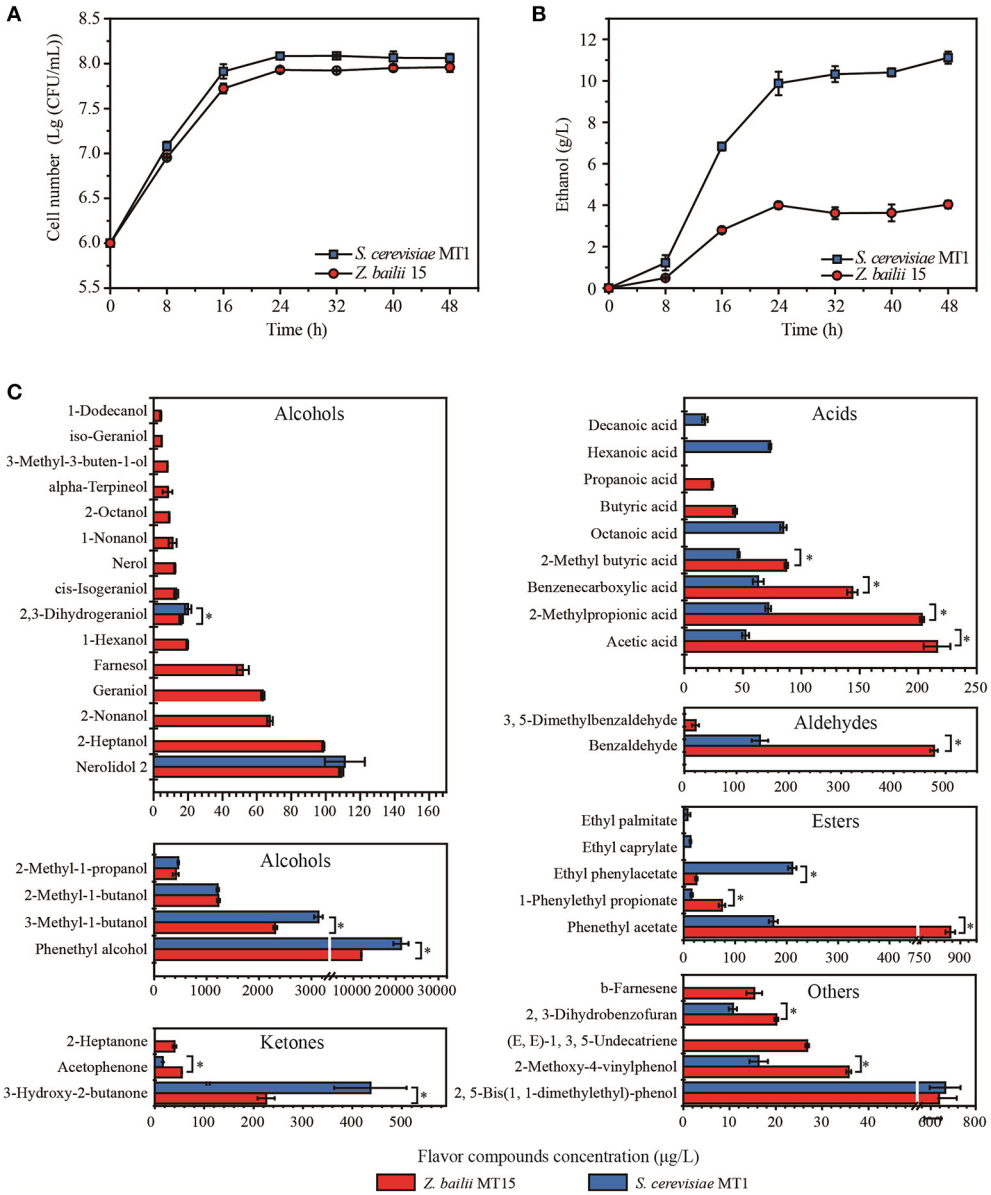
Biomass **(A)**, ethanol content **(B)**, and flavor compounds production **(C)** of *Z. bailii* MT15 and *S. cerevisiae* MT1. Significance level of the one-way ANOVA: ^*^P < 0.05.

*Z. bailii* MT15 and *S. cerevisiae* MT1 were pre-cultured in sorghum extract at 30°C for 16 h to obtain the seed culture. Then they were inoculated into fermentation media with an initial concentration of 1 × 10^6^ colony-forming units (CFU)/mL. For the comparison of fermentation characteristics between *Z. bailii* MT15 and *S. cerevisiae* MT1, the strains were, respectively, fermented at 30°C for 48 h, with shaking at 200 rpm. To study the effects of temperature on fermentation, *Z. bailii* MT15 was fermented at 30 and 37°C for 48 h, with shaking at 200 rpm. During the fermentation, 1 mL of samples were withdrawn at 8-h intervals to determine the cell numbers (Figure 1A). Each experiment was performed in triplicate.

### Analytical determinations and statistical analysis

Yeast cells in the seed culture and during the fermentation process were obtained by centrifugation, washed three times with sterile saline solution, diluted to the applicable concentration by saline solution, and counted by hemocytometer under microscope. To analyze the contents of ethanol, flavor compounds, and amino acids, fermentation broths were centrifuged at 8,000 × *g* for 10 min to remove cells. Ethanol was monitored by high-performance liquid chromatography (HPLC) with a refractive index detector (Varian 355 RI) (Meng et al., 2015). Flavor compounds were analyzed by the gas chromatography-mass spectrometry method (Kong et al., 2014). Free amino acids were detected by HPLC via a pre-column derivatization method (Zhang et al., 2014).

The data are presented in terms of arithmetic averages of three replicates and the error bars indicate the standard deviations. The statistical significance of the difference between the means of samples was tested by one-way analysis of variance (ANOVA) using SPSS Statistics 22.

### Genomic sequencing and assembly

The *Z. bailii* MT15 genome was sequenced using the whole-genome shotgun sequencing approach on an Illumina Miseq platform (Quail et al., 2012). Four paired-end/mate-paired sequencing libraries were constructed with insert sizes of 450, 700 bp, 3, and 8 kb. The *de novo* assembler Newbler and SSPACE software packages were employed to assemble raw data into contigs and scaffolds (Boetzer et al., 2011; Nederbragt, 2014). The GapCloser program was used to close gaps (Boetzer and Pirovano, 2012). The genome sequence of *Z. bailii* MT15 was deposited in GenBank under the Whole Genome Shotgun project number SRR5452526.

The genes of *Z. bailii* MT15 were predicted with the CEGMA pipeline, combining Augustus, SNAP, and Glimmer gene prediction software (Delcher et al., 1999; Stanke and Waack, 2003; Korf, 2004; Parra et al., 2007). The functional annotation of each gene was based on the eggNOG and Swissprot databases (Boeckmann et al., 2003; Powell et al., 2012). The functional classifications were performed with the eggNOG database. Genes for tRNAs and rRNAs were predicted with tRNAscan-SE and RNAmmer 1.2 Server, respectively (Schattner et al., 2005; Lagesen et al., 2007).

### cDNA preparation and transcriptome analysis using RNA sequencing

For RNA extraction, *Z. bailii* MT15 cells were cultivated with three parallel at 30 and 37, respectively, for 24 h. Then, the three parallel were mixed in the same volume and harvested after centrifugation at 8,000 × *g* for 5 min at 4°C. Subsequently, the supernatant was removed and the cell pellet was washed three times with sterile saline solution on ice. Then the washed cell pellet was immediately frozen in liquid nitrogen. Total RNA was isolated using the Trizol Reagent (Invitrogen Life Technologies, Shanghai) according to the manufacturer's instructions. Quality and integrity of total RNA were determined using a Nanodrop spectrophotometer (Thermo Scientific, USA) and Bioanalyzer 2100 system (Agilent Technologies, Santa Clara, CA). Ribo-Zero rRNA Removal Kit (Illumina, San Diego, CA) was used to remove the ribosomal RNAs. The mRNA was subsequently fragmented and used as a template for oligo (dT)-primed PCR.

The cDNA libraries were prepared employing standard techniques for subsequent Illumina sequencing using the mRNA-seq Sample Prep Kit (Illumina, San Diego, CA). The cDNA libraries were sequenced on an Illumina NextSeq 500 according to the manufacturer's instructions. Sequencing raw reads were pre-processed after filtering sequencing adapters, rRNA reads, short-fragment reads, and other low-quality reads. The remaining clear reads were mapped to the reference genome of *Z. bailii* MT15 using Bowtie2/Tophat2 software (Langmead and Salzberg, 2012; Kim et al., 2015) based on the local alignment algorithm. Gene expression level was normalized by calculating reads per kilobase per million reads (RPKM) (Mortazavi et al., 2008). Differential expression of all of transcripts was quantified using DESeq software, and the method of FDR (False Discovery Rate) control was used to correct the results for multiple hypothesis testing (Anders and Huber, 2013). Significant DEGs were screened based on an FDR threshold of ≤0.05, and a Fold change ≥1.5. The RNA sequence data of *Z. bailii* MT15 at 30 and 37°C was deposited in the DNA Data Bank of Japan (DDBJ) with accession IDs DRX082892 and DRX082893, respectively.

## Results and discussion

### Metabolic properties of *Z. bailii* MT15

The biomass and ethanol production of *Z. bailii* MT15 and *S. cerevisiae* MT1 was determined during the fermentation process. The cell number of *Z. bailii* MT15 and *S. cerevisiae* MT1 was 9.18 × 10^7^ and 8.18 × 10^7^ CFU/mL at the end of fermentation (Figure 1A), respectively, which showed no significantly statistical difference (Supplementary Table 1). The highest ethanol production of *Z. bailii* MT15 was 4.03 g/L, which was 63.76% less than that of *S. cerevisiae* MT1 (Figure 1B, Supplementary Table 2). Considering the large population of *Z. bailii* MT15, whose proportion can reach 78% of the total yeast population (Wu, 2013), we considered that this yeast also contributes to ethanol production in *Maotai*-flavor liquor fermentation.

Flavor compounds play essential roles in forming the unique flavor quality of *Maotai*-flavor liquor (Xu and Ji, 2012). The metabolic activity of *Z. bailii* MT15 in flavor production was studied at the end of fermentation and compared with that of *S. cerevisiae* MT1 (Figure 1C). Production of alcohols by *Z. bailii* MT15 was 16,333.78 μg/L, which was approximately half that of *S. cerevisiae* MT1. Production of ketones by *Z. bailii* MT15 was 319.95 μg/L, which was also less than that of *S. cerevisiae* MT1 (452.98 μg/L). However, the production of acids, esters, and aldehydes by *Z. bailii* MT15 was 1.75, 2.28, and 3.45 times of that of *S. cerevisiae* MT1, respectively.

Additionally, *Z. bailii* MT15 produced 38 flavor compounds including 19 alcohols, six acids, three esters, three ketones, two aldehydes, and five other compounds (Figure 1C). Among them, 19 flavor compounds were also detected in the fermentation broth of *S. cerevisiae* MT1. The amounts of 10 flavor compounds, including acetic acid, benzaldehyde, phenethyl acetate, 1-phenylethyl propionate, and acetophenone, produced by *Z. bailii* MT15 were significantly higher than those produced by *S. cerevisiae* MT1 (Figure 1C). Furthermore, *Z. bailii* MT15 was able to produce 19 unique flavor compounds compared with *S. cerevisiae* MT1, including 13 alcohols, two acids, one ketone, one aldehyde, and two other flavor compounds. Some of these unique flavor compounds were influential flavor compounds and contributed significantly to the flavor of the *Maotai*-flavor liquor, such as 2-heptanol (fruity flavor), 2-nonanol (fruity flavor), 1-nonanol (fruity flavor), 1-hexanol (floral, green scent), geraniol (sweet, rose-like scent), 2-heptanone (fruity, spicy, cinnamon scent), propionic acid (vinegar-like scent), and butyric acid (cheesy-like scent) (Xu and Ji, 2012).

These results demonstrated that *Z. bailii* MT15 could generate ethanol and various flavor compounds including alcohols, acids, esters, aldehydes, and ketones during liquor fermentation, which would contribute to the flavor and quality of *Maotai*-flavor liquor. Moreover, previous studies showed that *Z. bailii* contributed to the flavor complexity of wine and could be used as a mixed starter with *S. cerevisiae* to improve the production of ethyl esters in wine fermentation (Ciani et al., 2009; Garavaglia et al., 2015). Therefore, except for the capacity of flavor production, *Z. bailii* would interact with *S. cerevisiae* during liquor fermentation and positively affect the flavor and quality of *Maotai*-flavor liquor.

### Effect of temperature on flavor metabolism of *Z. bailii* MT15

*Maotai*-flavor liquor is produced by a spontaneous fermentation process with a relatively high temperature of up to about 37°C (Wu et al., 2013). However, little is known about the metabolic properties and mechanisms of *Z. bailii* under such a relatively high temperature in food fermentation. We therefore studied the metabolic activity of *Z. bailii* MT15 at 37°C, and compared it with that at 30°C.

**Figure 2:**
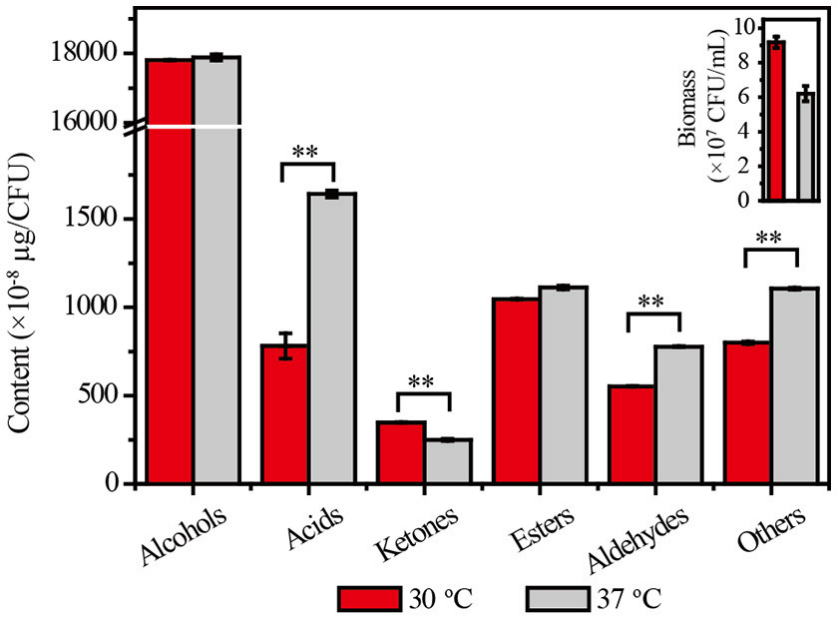
Cell growth and production of flavor compounds by *Z. bailii* MT15 at 30 and 37°C. Significance level of the one-way ANOVA: ^**^P < 0.01.

The biomass of *Z. bailii* MT15 decreased by 32.46% at 37°C compared with 30°C. Meanwhile, few differences were observed in the types of flavor compounds produced, but the amounts of flavor compounds per unit cell were substantially different between the two temperatures (Dataset 1). As shown in Figure 2, no significant change was observed in the production of alcohols and esters per unit cell, and the production of ketones decreased by 28.30% at the higher temperature. By contrast, the production of acids and aldehydes per unit cell at 37°C was, respectively, 110 and 41% higher than that at 30°C. *Maotai*-flavor liquor contains large amounts of acids and aldehydes, which have positive effects on its unique sensory characteristics (Fan et al., 2011; Xu et al., 2017). The results suggested that production of acids and aldehydes in *Z. bailii* MT15 was enhanced under higher temperature. This enhancement would probably contribute to the unique sensory characteristics of *Maotai*-flavor liquor. Nevertheless, the metabolic mechanisms for its vigorous activities under higher temperature remains unclear. Therefore, we used omics' technology including genomic and transcriptomic analysis to unravel the metabolic features and mechanisms of *Z. bailii* MT15.

### Genomic and transcriptomic analysis of *Z. bailii* MT15

The whole genome of *Z. bailii* MT15 was sequenced and compared with the genome of three other *Z. bailii* strains (*Z. bailii* CLIB 213^T^, *Z. bailii* ISA1307, and *Z. bailii* IST302) (Galeote et al., 2013; Mira et al., 2014; Palma et al., 2017), and the results are shown in Table 1. The *Z. bailii* MT15 genome assembly resulted in 287 contigs (>527 bp) with an N50 value of 156,420 bp, and 95 scaffolds (>2020 bp) with an N50 value of 684,448 bp. The assembled genome was 20.19 Mb with a GC content of 42.38 mol%. The genome size of *Z. bailii* MT15 was 1.97-fold that of *Z. bailii* CLIB 213^T^ and 1.87-fold that of *Z. bailii* IST302, but was similar to that of *Z. bailii* ISA1307. A total of 9,498 genes were predicted with an average length of 1,461 bp, occupying 68.72% of the whole genome (Table 1).

**Table 1:** General features of the *Z. bailii* MT15, *Z. bailii* CLIB 213^T^, *Z. bailii* ISA1307, and *Z. bailii* IST302 genomes.

**Feature**	***Z. bailii* MT15**	***Z. bailii* CLIB 213^T^**	***Z. bailii* ISA1307**	***Z. bailii* IST302**
Genome size (Mb)	20.19	10.27	21.14	10.77
GC content (mol%)	42.38	42.50	42.40	42.20
GC content in mRNA region (mol%)	–	–	43.80	NP
rRNA number	3	0	3	NP
tRNA number	507	162	514	NP
Coding sequence number	9498	4723	9925	5142
Average coding sequence length (bp)	1461	1475	1471	NP
Total coding sequence length (Mb)	13.88	6.97	14.76	NP
Coding sequence of genome (%)	68.72	67.97	69.80	NP

*NP, Not published*.

The number of genes in the *Z. bailii* MT15 genome was nearly twice that of *Z. bailii* CLIB 213^T^ and *Z. bailii* IST302, but was close to the number in *Z. bailii* ISA1307. It has been proven that *Z. bailii* ISA1307 is an interspecies hybrid strain that was generated in a stressful environment to improve strain robustness (Sipiczki, 2008; Mira et al., 2014). We analyzed the sequences of housekeeping genes including *RPB1, RPB2, TBB*, and *EFGM* in the *Z. bailii* MT15 genome, which are proposed to have a high capacity to discriminate *Zygosaccharomyces* species (Suh et al., 2013). The *RPB1, RPB2, TBB*, and *EFGM* genes were all duplicated in the *Z. bailii* MT15 genome, and the sequences of alleles of the four genes were different, which indicated that *Z. bailii* MT15 might be an interspecies hybrid strain. Furthermore, alleles of the four genes showed high sequence identity to the orthologous genes in *Z. bailii* CLIB 213^T^ and *Zygosaccharomyces parabailii* ATCC 60483 (Supplementary Table 3). Thus, it is likely that *Z. bailii* MT15 is an interspecies hybrid strain generated from *Z. bailii* and *Z. parabailii*, which was beneficial for its adaptation to the relatively high temperature environment in *Maotai*-flavor liquor fermentation.

**Figure 3:**
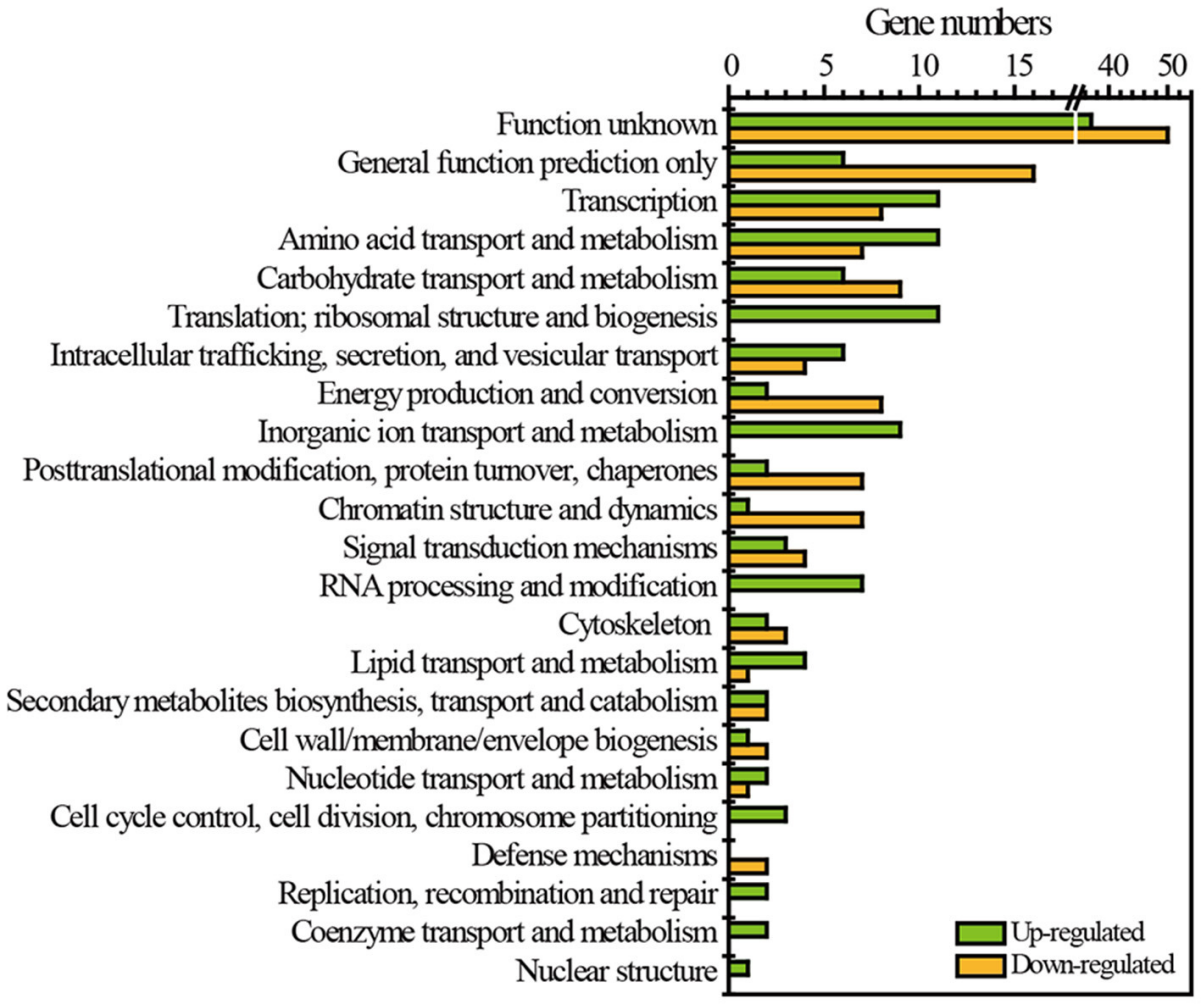
Functional classes of DEGs of *Z. bailii* MT15 at 30 and 37°C using eggNOG functional categories.

RNA-Seq was employed to reveal the transcriptomic features of flavor metabolism in *Z. bailii* MT15 under heat stress at 30and 37°C. The results showed that 257 genes (2.71% of the *Z. bailii* MT15 genome) were differentially expressed (≥1.5-fold, *P* < 0.05), including 126 up-regulated genes (Dataset 2) and 131 down-regulated genes (Dataset 3). These differentially expressed genes (DEGs) were clustered according to eggNOG functional categories (Figure 3). Among these categories, general function prediction only (8.56% of DEGs), transcription (7.39% of DEGs), amino acid transport and metabolism (7.00% of DEGs), and carbohydrate transport and metabolism (5.84% of DEGs), contained the greatest number of DEGs (Figure 3). Amino acid metabolism is important for the production of ethanol and most flavor compounds in liquor; therefore, we further analyzed the DEGs involved in amino acid transport and metabolism.

**Figure 4:**
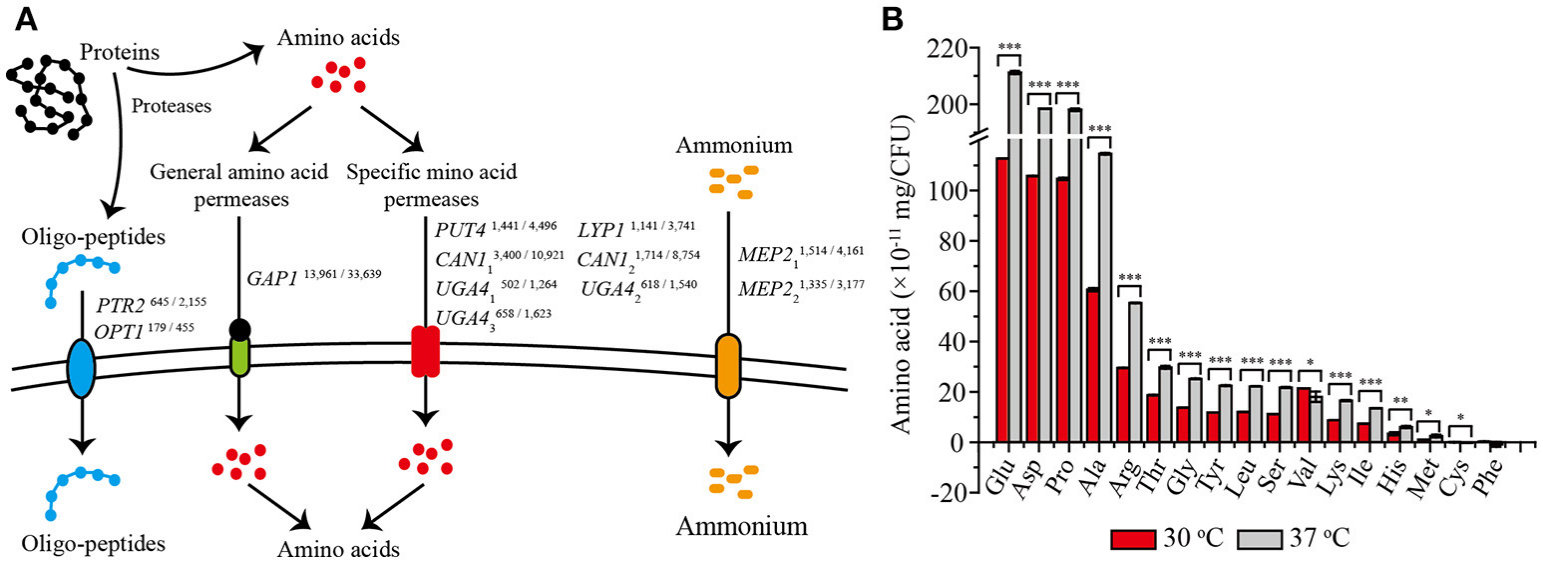
Differentially expressed genes related to amino acid transport **(A)** and the absorption of 17 conventional amino acids **(B)** at 30 and 37°C after 24 h of cultivation. The RPKM value of each gene is presented in the upper right corner, and the numbers on the left and right represent the RPKM values of *Z. bailii* MT15 at 30 and 37°C, respectively. Significance level of the one-way ANOVA: ^*^0.05 > *P* > 0.01; ^**^0.01 > *P* > 0.001; ^***^P < 0.001.

Comparative transcriptome analysis showed that 12 genes related to amino acid transport and metabolism were significantly up-regulated at 37°C (Figure 4A). Among them, eight genes encoded amino acid permeases, including one general amino acid permease and seven permeases specific for arginine, proline, lysine, and γ-aminobutyric acid. Higher transcription levels of these genes would be important for the transport of more amino acids into yeast cells (Jauniaux and Grenson, 1990; Regenberg et al., 1999). The other four genes were found to be involved in amino acids biosynthesis, including *PTR2* and *OPT1* associated with the transport of small peptides, and *MEP2*_1_ and *MEP2*_2_ encoding ammonium transporters, would also contribute to a strengthened amino acid metabolism (Meister, 1957). Thus, we speculated that these up-regulated amino acid metabolism related genes would promote the absorption and utilization of amino acid at 37°C. To validate this speculation, we detected the absorption of the most conventional amino acids by *Z. bailii* MT15 at 30 and 37°C after 24 h of cultivation. As shown in Figure 4B, the absorption of 14 amino acids was obviously improved by 83% at 37°C, including the uptake of glutamate acid, aspartate, proline, alanine, and arginine. Among these, the uptake of glutamate acid increased most prominently from 1.14 × 10^−9^ to 2.11 × 10^−9^ mg/CFU. By contrast, the absorption of valine and cysteine decreased, and no significant difference was found in the phenylalanine absorption. Therefore, we can conclude that *Z. bailii* MT15 could absorb and utilize amino acids more efficiently at 37 than at 30°C, due to the strengthened amino acid associated pathway at higher temperature. The strengthened absorption and utilization of amino acids possibly contributed to the improvement of the corresponding flavor compounds production, in particular, the production of acids and aldehydes.

### Metabolic mechanisms of production of flavor compounds in *Z. bailii* MT15

**Figure 5:**
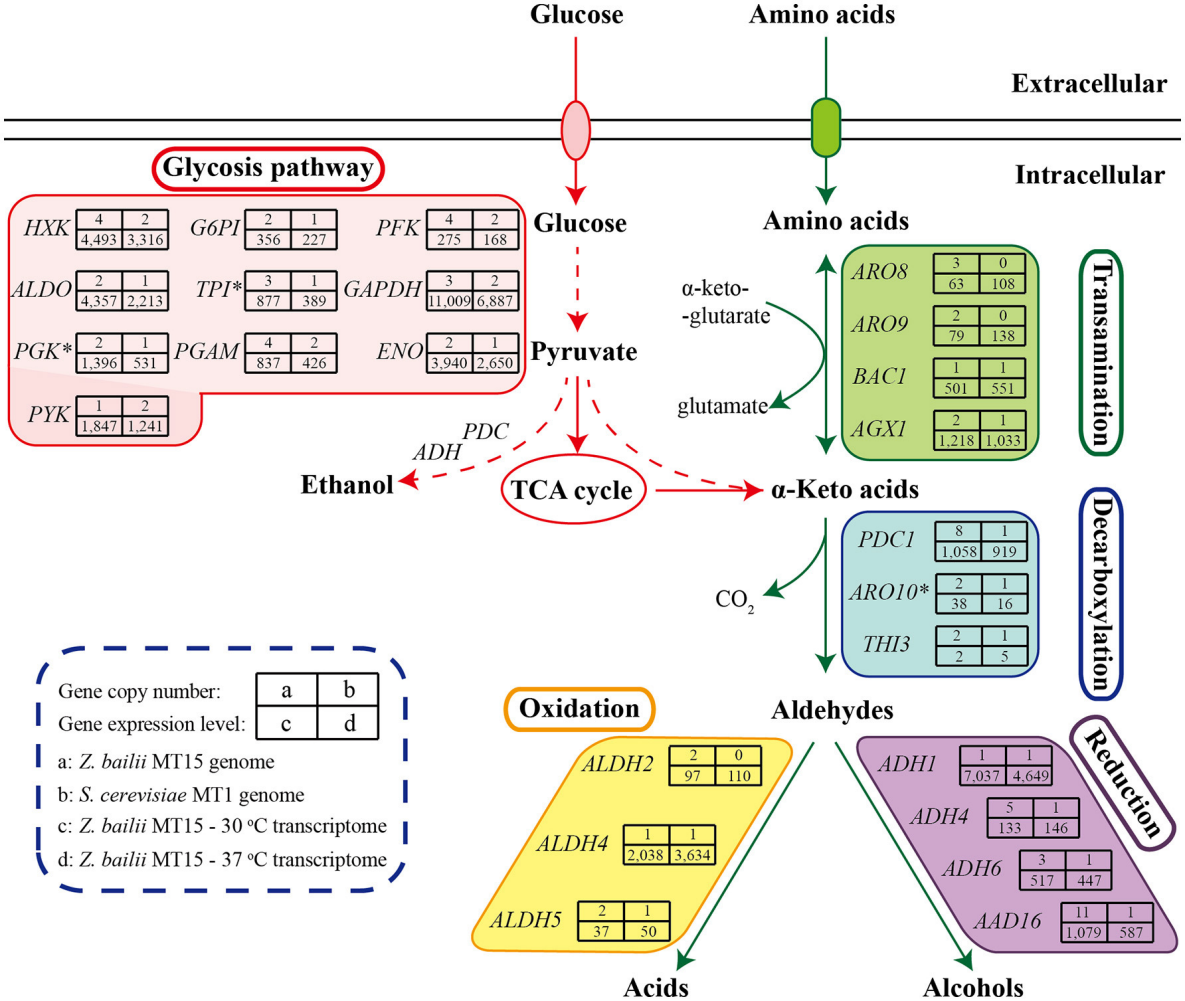
Genome and transcriptome analysis of *Z. bailii* MT15 in flavor production. Catabolism of sugars and amino acids leads to the formation of various flavor compounds. Genes encoding the key enzymes required in each step are indicated. ^*^This gene was a DEG (≥2-fold, *P* < 0.05) at 37°C.

*Z. bailii* MT15 could produce various flavor compounds, especially alcohols, acids, and esters (Figure 2). During food fermentation, alcohols and acids are generated by yeasts from amino acids through the Ehrlich pathway and from sugars through the Harris pathway (Figure 5; Ehrlich, 1907; Chen, 1978). To unravel the molecular mechanisms of flavor metabolism in *Z. bailii* MT15, we compared its genome with that of strain *S. cerevisiae* MT1 and analyzed the transcription levels of genes involved in flavor metabolism at 37 and 30°C.

As shown in Figure 5, sugars could be converted to α-keto acids via the glycolysis and tricarboxylic acid (TCA) cycle pathways. In the *Z. bailii* MT15 genome, the copy number of genes involved in glycolysis and the TCA cycle was around twice those in the *S. cerevisiae* MT1 genome (Supplementary Table 4), which was expected as *Z. bailii* MT15 was considered to be an interspecies hybrid strain. In addition, α-keto acids could also be generated by various aminotransferases through the Ehrlich pathway (Hazelwood et al., 2008). In the *Z. bailii* MT15 genome, at least 16 different genes were annotated as aminotransferases including *AGX1, BCA1, ARO9, ARO8*, and *YGD3*. (Supplementary Table 5). Among them, *ARO9* and *ARO8* were uniquely found in the *Z. bailii* MT15 genome and might be associated with the differences in flavor metabolism compared with *S. cerevisiae* MT1.

The α-keto acids were further decarboxylated to the corresponding aldehydes by α-keto acid decarboxylases including *PDC1, PDC5, PDC6, ARO10*, and *THI3* (ter Schure et al., 1998; Dickinson et al., 2003; Vuralhan et al., 2005). Among them, *PDC1, ARO10*, and *THI3* existed in both of the two strains, while *PDC5* and *PDC6* were uniquely found in the *S. cerevisiae* MT1 genome. Pyruvate decarboxylases (PDCs) participate in alcoholic fermentation by converting pyruvate to acetaldehyde and in amino acid metabolism through the Ehrlich pathway (ter Schure et al., 1998). We found 13 genes encoding PDC enzymes in the *Z. bailii* MT15 genome, whereas only six were found in the *S. cerevisiae* MT1 genome. Since the ethanol production of *Z. bailii* MT15 was less than that of *S. cerevisiae* MT1 (Figure 1B), more PDC genes in *Z. bailii* MT15 did not favor its alcoholic fermentation, but might be beneficial for the conversion of amino acids and reducing sugars to aldehydes by the Ehrlich pathway.

Aldehydes could subsequently be converted to higher alcohols and acids by alcohol dehydrogenases and aldehyde dehydrogenases, respectively (Hazelwood et al., 2008). So far, at least 16 genes encoding alcohol dehydrogenases have been found to catalyze the interconversion of aldehydes and alcohols (Hazelwood et al., 2008). However, only four of these genes (*ADH1, ADH4, ADH6*, and *AAD16*) were found in the *Z. bailii* MT15 genome, while 11 such genes were present in the *S. cerevisiae* MT1 genome. *Z. bailii* MT15 harboring fewer alcohol dehydrogenases genes may be the reason that it produced more aldehydes and less alcohols compared with *S. cerevisiae* MT1. Moreover, the genome analysis showed that three genes (*ALDH2, ALDH4*, and *ALDH5*) annotated as aldehyde dehydrogenase were found in the *Z. bailii* MT15 genome, and *ALDH2* was a specific gene compared with *S. cerevisiae* MT1. This would account for the higher acid productivity of *Z. bailii* MT15. Therefore, we can conclude that genome differences, particularly in the aspect of genes involved in amino acid metabolism between *Z. bailii* MT15 and *S. cerevisiae* MT1, would lead to the different metabolic features of the two strains (Figure 1C).

The transcriptome comparison of *Z. bailii* MT15 revealed that genes involved in the glycolysis pathway showed lower RPKM values at 37 than those at 30°C, which indicated that the glycolysis pathway might not be related to the increased production of acids and aldehydes at higher temperature. Moreover, in the TCA cycle, *ACON2* associated with the production of α-ketoglutaric acid was significantly up-regulated at 37°C, which would promote production of α-keto acids. Meanwhile, the RPKM values of *ARO8* and *ARO9*, which are involved in the transamination step of the Ehrlich pathway and regarded as broad-substrate-specificity aminotransferases (Iraqui et al., 1998), were higher at 37 than those at 30°C (≥1.50-fold, *P* ≤ 0.25). The higher expression of these genes might be beneficial for the transamination of amino acids to corresponding α-keto acids at 37°C. Furthermore, *ALDH4* also showed higher RPKM values at 37°C (1.78-fold, *P* = 0.19), which was consistent with the increased production of acids at 37°C (Figure 2). Therefore, the higher expression of genes, including *ACON2, ARO8, ARO9*, and *ALDH4* in the Harris pathway and Ehrlich pathway, might be beneficial for the increased production of acids and aldehydes at the relatively high fermentation temperature.

## Conclusions

This study revealed the fermentation characteristics and potential function of *Z. bailii* MT15 that produces various flavor compounds including alcohols, acids, esters, aldehydes, and ketones. Its ability to generate acids and aldehydes is improved at the relatively high temperature used in liquor fermentation, which is beneficial for the complexity of the aroma and quality of the liquor. The genome and transcriptome analysis of *Z. bailii* MT15 revealed that amino acids metabolism plays important roles in flavor production. This work sheds new light on the metabolic characteristics of *Z. bailii* in flavor production during *Maotai*-flavor liquor fermentation that would be applicable to various food fermentations.

## Author contributions

YX (first author), QW, and YZ drafted the manuscript. YX (first author), QW, and RD performed the physiological studies and genome sequencing and transcriptome analysis. QW and YX (last author) participated in the design of the study. All authors read and approved the final manuscript.

### Conflict of interest statement

The authors declare that the research was conducted in the absence of any commercial or financial relationships that could be construed as a potential conflict of interest.
