# Supplementary material for: Zygosaccharomyces bailii Is a Potential Producer of Various Flavor Compounds in Chinese Maotai-Flavor Liquor Fermentation
Source: Front Microbiol. 2017 Dec 22;8:2609. doi: 10.3389/fmicb.2017.02609 (PMC5744019; doi:10.3389/fmicb.2017.02609)
Supplement: Supplementary file 1 [file Table1.DOCX]

**Supplementary Table 1 Cell number in fermentation process**

| Time(h) | Log cell number (mean±SD) | |
| --- | --- | --- |
|  | *Z. bailii* MT15 | *S. cerevisiae* MT1 |
| 0 | 6.00±0.00^a^ | 6.00±0.00^a^ |
| 8 | 6.95±0.02^a^ | 7.08±0.05^b^ |
| 16 | 7.72±0.05^a^ | 7.91±0.07^b^ |
| 24 | 7.93±0.03^a^ | 8.08±0.04^b^ |
| 32 | 7.92±0.01^a^ | 8.09±0.01^b^ |
| 40 | 7.95±0.03^a^ | 8.06±0.06^a^ |
| 48 | 7.96±0.05^a^ | 8.01±0.04^a^ |

^a,b^ Values with different letters in a row indicate that they are significantly different from each other (P < 0.05)
