# Supplementary material for: Zygosaccharomyces bailii Is a Potential Producer of Various Flavor Compounds in Chinese Maotai-Flavor Liquor Fermentation
Source: Front Microbiol. 2017 Dec 22;8:2609. doi: 10.3389/fmicb.2017.02609 (PMC5744019; doi:10.3389/fmicb.2017.02609)
Supplement: Supplementary file 2 [file Table2.DOCX]

**Supplementary Table 2 Ethanol content in fermentation process**

| Time(h) | Ethanol content g/L (mean ± SD) | |
| --- | --- | --- |
|  | *Z. bailii* MT15 | *S. cerevisiae* MT1 |
| 0 | 0.00±0.00^a^ | 0.00±0.00^a^ |
| 8 | 0.49±0.03^a^ | 1.22±0.37^b^ |
| 16 | 2.79±0.15^a^ | 6.83±0.20^b^ |
| 24 | 3.99±0.16^a^ | 9.88±0.54^b^ |
| 32 | 3.61±0.29^a^ | 10.32±0.39^b^ |
| 40 | 3.63±0.38^a^ | 10.41±0.20^b^ |
| 48 | 4.03±0.19^a^ | 11.12±0.27^b^ |

^a, b^ Values with different letters in a row indicate that they are significantly different from each other (P < 0.05)
