# Supplementary material for: Zygosaccharomyces bailii Is a Potential Producer of Various Flavor Compounds in Chinese Maotai-Flavor Liquor Fermentation
Source: Front Microbiol. 2017 Dec 22;8:2609. doi: 10.3389/fmicb.2017.02609 (PMC5744019; doi:10.3389/fmicb.2017.02609)
Supplement: Supplementary file 3 [file Table3.DOCX]

**Supplementary Table 3 Molecular typing of *Z. bailii* MT15**

| Gene | Top similar hits | Nucleotide variations |
| --- | --- | --- |
| *RPB1_1* (scaffold5.g283) | *Z. bailii* CLIB 213T | 5211/5214(99%) |
|  | *Z. parabailii* ATCC 60483 | 5168/5171(99%) |
|  | *Z. kombuchaensis* CBS 8849 | 4273/5202(82%) |
| *RPB1_2* (scaffold7.g281) | *Z. parabailii* ATCC 60483 | 4810/5141(94%) |
|  | *Z. bailii* CLIB 213T | 4808/5141(94%) |
|  | *Z. kombuchaensis* CBS 8849 | 4202/5144(82%) |
| *RPB2_1* (scaffold10.g162) | *Z. bailii* CLIB 213T | 3670/3672(99%) |
|  | *Z. parabailii* ATCC 60483 | 3668/3672(99%) |
|  | *Z. kombuchaensis* CBS 8849 | 3081/3673(84%) |
| *RPB2_2* (scaffold11.g188) | *Z. parabailii* ATCC 60483 | 3471/3672(95%) |
|  | *Z. bailii* CLIB 213T | 3468/3672(94%) |
|  | *Z. kombuchaensis* CBS 8849 | 3079/3672(84%) |
| *TBB_1* (scaffold5.g409) | *Z. bailii* CLIB 213T | 1341/1341(100%) |
|  | *Z. parabailii* ATCC 60483 | 1340/1341(99%) |
|  | *Z. parabailii* ATCC 56075 | 846/882(96%) |
| *TBB_2* (scaffold7.g407) | *Z. parabailii* ATCC 60483 | 1283/1341(96%) |
|  | *Z. bailii* CLIB 213T | 1280/1341(95%) |
|  | *Z. pseudobailii* ATCC 56074 | 840/883(95%) |
| *EFGM_1* (scaffold5.g419) | *Z. parabailii* ATCC 60483 | 2288/2289(99%) |
|  | *Z. bailii* CLIB 213T | 2287/2289(99%) |
|  | *Z. rouxii* CBS732 | 1723/2135(81%) |
| *EFGM_2* (scaffold7.g417) | *Z. parabailii* ATCC 60483 | 2147/2287(94%) |
|  | *Z. bailii* CLIB 213T | 2146/2287(94%) |
|  | *Kluyveromyces lactis* NRRL Y-1140 | 1636/2090(78%)^*^ |

The two gene copies of *RPB1*, *RPB2*, *TBB* and *EFGM* found in the *Z. bailii* MT15 genome were named 1 and 2. ‘^*^’: There was 1% gaps of the nucleotide sequences of the *EFGM_2* gene in the *Z. bailii* MT15 and *K. lactis* NRRL Y-1140 genomes.
