# Supplementary material for: Zygosaccharomyces bailii Is a Potential Producer of Various Flavor Compounds in Chinese Maotai-Flavor Liquor Fermentation
Source: Front Microbiol. 2017 Dec 22;8:2609. doi: 10.3389/fmicb.2017.02609 (PMC5744019; doi:10.3389/fmicb.2017.02609)
Supplement: Supplementary file 4 [file Table4.DOCX]

**Supplementary Table 4 Analysis of genes involved in the synthesis of α-ketoglutaric acids**

| Genes | Function | *Z. bailii* MT15 | *S. cerevisiae* MT1 |
| --- | --- | --- | --- |
| *CIT* | Citrate synthase | scaffold2.g70 | maker-contig21-snap-gene-0.79-mRNA-1 |
|  |  | scaffold12.g244 | augustus-contig42-abinit-gene-0.39-mRNA-1 |
|  |  | scaffold5.g340 | maker-contig7-snap-gene-0.942-mRNA-1 |
|  |  | scaffold7.g338 |  |
| *ACON* | Aconitase | scaffold10.g43 | augustus-contig34-abinit-gene-0.141-mRNA-1 |
|  |  | scaffold11.g309 | augustus-contig16-abinit-gene-0.57-mRNA-1 |
| *IDH* | Isocitrate dehydrogenase | scaffold2.g46 | augustus-contig20-abinit-gene-0.4-mRNA-1 |
|  |  | scaffold12.g267 | augustus-contig21-abinit-gene-0.24-mRNA-1 |
|  |  | scaffold4.g285 | maker-contig13-augustus-gene-0.464-mRNA-1 |
|  |  | scaffold8.g286 | augustus-contig40-abinit-gene-0.135-mRNA-1 |
|  |  | scaffold10.g138 |  |
|  |  | scaffold11.g212 |  |
|  |  | scaffold17.g131 |  |
|  |  | scaffold18.g66 |  |
