# Supplementary material for: Zygosaccharomyces bailii Is a Potential Producer of Various Flavor Compounds in Chinese Maotai-Flavor Liquor Fermentation
Source: Front Microbiol. 2017 Dec 22;8:2609. doi: 10.3389/fmicb.2017.02609 (PMC5744019; doi:10.3389/fmicb.2017.02609)
Supplement: Supplementary file 5 [file Table5.DOCX]

| **Supplementary Table 5 Genes annotated as aminotransferases in the *Z. bailii* MT15** | | | | |
| --- | --- | --- | --- | --- |
| Gene ID | Name | Function | RPKM-30 ^o^C | RPKM-37 ^o^C |
| scaffold5.g399 | *AGX1* | Alanine--glyoxylate aminotransferase 1 | 1218.33 | 1032.69 |
| scaffold7.g397 | *AGX1* | Alanine--glyoxylate aminotransferase 1 | 1155.71 | 730.62 |
| scaffold39.g16 | *BCA1* | Branched-chain-amino-acid aminotransferase, mitochondrial | 500.89 | 550.65 |
| scaffold9.g332 | *ARO9* | Aromatic amino acid aminotransferase 2 | 92.13 | 134.60 |
| scaffold14.g232 | *ARO9* | Aromatic amino acid aminotransferase 2 | 79.03 | 138.17 |
| scaffold25.g3 | *YGD3* | Uncharacterized aminotransferase C1771.03c | 67.82 | 34.65 |
| scaffold9.g173 | *YGD3* | Uncharacterized aminotransferase C1771.03c | 67.35 | 56.81 |
| scaffold24.g125 | *ARO8* | Aromatic/aminoadipate aminotransferase 1 | 63.07 | 108.25 |
| scaffold1.g335 | *YGD3* | Uncharacterized aminotransferase C1771.03c | 52.97 | 59.87 |
| scaffold21.g9 | *ARO8* | Aromatic/aminoadipate aminotransferase 1 | 48.16 | 71.66 |
| scaffold22.g75 | *ALAM* | Probable alanine aminotransferase, mitochondrial | 25.70 | 40.63 |
| scaffold14.g154 | *ALAM* | Probable alanine aminotransferase, mitochondrial | 18.23 | 34.02 |
| scaffold1.g202 | *GABAT* | 4-aminobutyrate aminotransferase | 16.29 | 18.73 |
| scaffold9.g141 | *AATC* | Aspartate aminotransferase, cytoplasmic | 15.25 | 16.79 |
| scaffold30.g85 | *YGD3* | Uncharacterized aminotransferase C1771.03c | 14.80 | 6.05 |
| scaffold4.g222 | *OAT* | Ornithine aminotransferase | 14.78 | 15.72 |
| scaffold1.g303 | *AATC* | Aspartate aminotransferase, cytoplasmic | 12.71 | 14.52 |
| scaffold3.g188 | *GFA1* | Glutamine--fructose-6-phosphate aminotransferase [isomerizing] | 12.54 | 21.10 |
| scaffold11.g71 | *SERC* | Phosphoserine aminotransferase | 11.92 | 14.66 |
| scaffold2.g461 | *HIS8* | Histidinol-phosphate aminotransferase | 11.53 | 8.49 |
| scaffold51.g3 | *ARO8* | Aromatic/aminoadipate aminotransferase 1 | 11.47 | 8.43 |
| scaffold14.g47 | *ARGD* | Acetylornithine aminotransferase, mitochondrial | 10.87 | 6.10 |
| scaffold33.g28 | *ARGD* | Acetylornithine aminotransferase, mitochondrial | 10.73 | 7.48 |
| scaffold9.g38 | *GABAT* | 4-aminobutyrate aminotransferase | 10.36 | 13.93 |
| scaffold8.g220 | *OAT* | Ornithine aminotransferase | 10.05 | 9.67 |
| scaffold41.g7 | *YHKC* | Uncharacterized aminotransferase C660.12c | 9.06 | 7.77 |
| scaffold6.g166 | *GFA1* | Glutamine--fructose-6-phosphate aminotransferase [isomerizing] | 8.48 | 16.96 |
| scaffold41.g3 | *YGD3* | Uncharacterized aminotransferase C1771.03c | 8.33 | 8.61 |
| scaffold8.g49 | *HIS8* | Histidinol-phosphate aminotransferase | 5.18 | 1.60 |
| scaffold6.g168 | *AATM* | Aspartate aminotransferase, mitochondrial | 5.15 | 3.31 |
| scaffold3.g190 | *AATM* | Aspartate aminotransferase, mitochondrial | 3.73 | 3.85 |
| scaffold29.g85 | *YGD3* | Uncharacterized aminotransferase C1771.03c | 3.44 | 1.79 |
| scaffold23.g106 | *YGD3* | Uncharacterized aminotransferase C1771.03c | 3.02 | 2.10 |
| scaffold10.g280 | *SERC* | Phosphoserine aminotransferase | 2.51 | 3.84 |
| scaffold3.g511 | *AAT* | Aspartate aminotransferase | 0.62 | 0.63 |
| scaffold24.g8 | *AAT* | Aspartate aminotransferase | 0.56 | 0.49 |
| scaffold5.g447 | *YGD3* | Uncharacterized aminotransferase C1771.03c | 0.41 | 0.25 |
| scaffold7.g446 | *YGD3* | Uncharacterized aminotransferase C1771.03c | 0.21 | 0.04 |
